# Surfactant Protein D Contributes to Ocular Defense against *Pseudomonas aeruginosa* in a Murine Model of Dry Eye Disease

**DOI:** 10.1371/journal.pone.0065797

**Published:** 2013-06-06

**Authors:** Susan R. Heimer, David J. Evans, James J. Mun, Michael E. Stern, Suzanne M. J. Fleiszig

**Affiliations:** 1 School of Optometry, University of California, Berkeley, California, United States of America; 2 College of Pharmacy, Touro University-California, Vallejo, California, United States of America; 3 Allergan Inc., Irvine, California, United States of America; 4 Graduate Groups in Vision Science, Microbiology, and Infectious Diseases and Immunity, University of California, Berkeley, California, United States of America; University of Florida, United States of America

## Abstract

Dry eye disease can cause ocular surface inflammation that disrupts the corneal epithelial barrier. While dry eye patients are known to have an increased risk of corneal infection, it is not known whether there is a direct causal relationship between these two conditions. Here, we tested the hypothesis that experimentally-induced dry eye (EDE) increases susceptibility to corneal infection using a mouse model. In doing so, we also examined the role of surfactant protein D (SP-D), which we have previously shown is involved in corneal defense against infection. Scopolamine injections and fan-driven air were used to cause EDE in C57BL/6 or Black Swiss mice (wild-type and SP-D gene-knockout). Controls received PBS injections and were housed normally. After 5 or 10 days, otherwise uninjured corneas were inoculated with 10^9^ cfu of *Pseudomonas aeruginosa* strain PAO1. Anesthesia was maintained for 3 h post-inoculation. Viable bacteria were quantified in ocular surface washes and corneal homogenates 6 h post-inoculation. SP-D was measured by Western immunoblot, and corneal pathology assessed from 6 h to 4 days. EDE mice showed reduced tear volumes after 5 and 10 days (each by ∼75%, p<0.001) and showed fluorescein staining (i.e. epithelial disruption). Surprisingly, there was no significant difference in corneal pathology between EDE mice and controls (∼10–14% incidence). Before bacterial inoculation, EDE mice showed elevated SP-D in ocular washes. After inoculation, fewer bacteria were recovered from ocular washes of EDE mice (<2% of controls, p = 0.0004). Furthermore, SP-D knockout mice showed a significant increase in *P. aeruginosa* corneal colonization under EDE conditions. Taken together, these data suggest that SP-D contributes to corneal defense against *P. aeruginosa* colonization and infection in EDE despite the loss of barrier function to fluorescein.

## Introduction

Bacterial keratitis is a severe, vision-threatening disease of the cornea associated with contact lens wear or ocular injury [1]. To this end, bacterial keratitis research has mostly focused on contact lens-wearing patient populations [2], or involved animal models of keratitis in which the cornea is either scratch-injured to allow infection or less commonly fitted with a contact lens [3]–[6]. These types of studies have helped identify numerous bacterial and host immune events that are important for disease pathogenesis, and have highlighted the resilience of the healthy ocular surface against infection. While other ocular surface diseases have also been associated with microbial keratitis, e.g. keratopathies [7] or dry eye diseases [8], little is known of the mechanisms involved.

The estimated prevalence of dry eye disease among microbial keratitis cases varies with study design, ranging from 7–15% in patients seeking treatment in a hospital or eye clinic setting [8]–[10], and up to 26% of patients dwelling in convalescent homes [11], [12]. Causative agents are mostly well-recognized opportunistic ocular pathogens such as coagulase-negative *Staphylococcus* spp., *S. aureus*, *Corynebacterium* spp. *Streptococcus pneumoniae*, and *Pseudomonas aeruginosa* [11]. Specific changes in the tear film composition have been reported that suggest dry eye disease patients may be compromised in defenses against microbial colonization. For example, a hallmark of dry eye inflammation in Sjögren's Syndrome is the depletion of conjunctival goblet cells which normally produce copious amounts of a gel-forming mucins MUC5A and MUC19 [13], [14], which trap bacteria and facilitate their clearance [15]. Dry eye patient tear samples also have been reported to differ in the relative abundance of antimicrobial factors including lysozyme, lactoferrin, lipocalin, MUC1, MUC4, MUC16, and beta-defensins [16]–[21]. Proinflammatory cytokines, e.g. IL-1β, are elevated in patients with dry eye disease as are matrix metalloproteinases such as MMP-9 [22]. Similar results have been obtained in experimentally-induced dry eye (EDE) animal models [23], [24], and associated with changes in the structural integrity of the corneal epithelium [25], [26]. More recently, the proinflammatory cytokine IL-17 was shown to be important in the pathogenesis of EDE [27], [28]. Recent studies have also shown an upregulation of secretory phospholipase A2 (sPLA2-IIa), an inflammatory disease biomarker and mediator, in patients with dry eye disease and in EDE [29], [30]. However, it is not yet known if one or more of these tear and corneal epithelial changes associated with dry eye disease or EDE predispose the cornea to infection.

Several of our previous studies using *P. aeruginosa* have highlighted the importance of tear fluid in protecting the cornea from infection. These include direct effects of tear fluid on bacteria, preventing invasion, cytotoxicity and epithelial traversal [31], [32], and indirect effects of tears by induction of corneal epithelial antimicrobial and immunomodulatory factors, e.g. RNase7 and ST-2 [33]. Our other previous studies have also shown the importance of surfactant protein-D, found in tear fluid and the corneal epithelium, in helping the ocular surface defend against *P. aeruginosa* and its pathogenic mechanisms [34]–[36]. Here, we tested the hypothesis that EDE would alter corneal susceptibility to *P. aeruginosa* colonization and infection in vivo. Our results showed that the murine cornea retained its resistance to *P. aeruginosa* infection under EDE conditions, and part of that resistance was associated with the increased expression of SP-D.

## Materials and Methods

### Ethics Statement

All procedures involving animals were carried out in accordance with standards established by the Association for the Research in Vision and Ophthalmology, and under a protocol approved by the Animal Care and Use Committee, University of California, Berkeley, an AAALAC accredited institution.

### Experimentally-Induced Dry Eye (EDE) Murine Model

EDE was induced in female, 6–8 weeks old C57BL/6 mice (Charles River Laboratories, Boston, MA), or in female or male 6–8 weeks old SP-D gene knockout (*sp-d* −/−) Black Swiss mice (a generous gift of Dr. Samuel Hawgood, University of California, San Francisco) along with strain/age/sex-matched controls (Taconic Farms, Cambridge City, IN). Mice were given subcutaneous injections of scopolamine hydrobromide (0.2 mL of 2.5 mg/mL per 20 g body weight; Sigma-Aldrich, St. Louis, MO) 3 times daily, alternating between right and left flanks, as previously described [37]. Animals were housed in mesh-sided cages, exposed to continuous fan-generated air drafts of low humidity (35–40%) for a period of 5 or 10 days. Control mice received vehicle only (PBS) injections and were housed under standard vivarium conditions without air drafts and normal humidity (40–50%). Aqueous tear production was assessed by placing a cotton thread (Zone Quick; FCI Ophthalmics, Marshfield, MA) in the lateral canthus for 30 s as previously described [26], and was reported as millimeters of wetted thread. When appropriate animals were anesthetized with intraperitoneal injections of 1.5 mg ketamine, 0.17 mg xylazine, 21 µg acepromazine per 20 g body weight. All mice (C57BL/6 or Black Swiss) developed similar levels of EDE. At the conclusion of each experiment, tissue samples were collected from euthanized animals.

### Fluorescein Staining

The corneas of anesthetized mice were topically infused with 3 µL of a sterile sodium fluorescein suspension (100 mL PBS rinse of a Fluoret stick; Chavvin, Aubenas, FR) for 3 min. Excess fluorescein was removed by washing with 1 mL of PBS. Corneal staining was observed under 20× magnification with a dissecting stereomicroscope (Zeiss, Jena, Germany) equipped with a blue light illumination, and documented with a AxioCam MR (Zeiss, Jena, Germany).

### Bacterial inoculation and quantification

*P. aeruginosa* strain PAO1 (serogroup O5) was used for this study. PAO1 is able to invade corneal epithelial cells and is virulent in a scarified murine cornea [38]. Bacteria were grown on Trypticase soy agar (TSA) at 37°C for 16 h and then resuspended in sterile phosphate-buffered saline (PBS) to a concentration of 10^11^ cfu/mL. Bacterial concentrations were confirmed by quantitative plating on TSA for viable counts. Following 5 or 10 day course of EDE induction or control treatments, ocular surfaces of anesthetized mice were inoculated topically with 5 µL containing 10^9^ cfu bacteria without introducing mechanical injury. Mice were maintained under sedation for the initial phase of the challenge ∼3 h. At various times after inoculation, viable bacteria in tear fluids or corneal tissues were assessed using quantitative plating on TSA [36].

### Ocular Surface Washes, Corneal Homogenates and Determination of Ocular Pathology

Murine tear fluids were harvested by washing the ocular surface of anesthetized mice with 5 µL of sterile PBS and collecting the washes with sterile, glass microcapilliary tubes (10 µL; Drummond Scientific Inc, Broomall, PA) placed in the lateral canthus. These ocular surface washes (2 µL) were serially diluted and plated for viable bacteria. To prepare corneal homogenates, eyes were collected from euthanized animals, corneal tissues were harvested ex situ and washed extensively with PBS (10 mL). The corneas were homogenized in 100 µL PBS containing 0.25% Triton X-100 with sterile Kontes microtube pellet pestle (Daigger, Vernon Hills, IL) and sampled for viable bacteria. Corneal pathology was assessed at various times pre- and post-inoculation, and documented with a digital CCD camera (Optronics, Goleta, CA) stereomicroscope system (Stemi 2000-C; Carl Zeiss, Thornwood, NY). Pathology was scored on 5-point grading system (0–4) based on the amount of surface area involved, the density of an opacity, and the overall surface regularity similar to that previously described [38]. Scores ranged from 0 (no infection) to a maximum of 12 (severe infection).

### SP-D Detection

SP-D in murine tear fluids was detected by Western Immunoblot as described previously [35]. Tear fluids were collected by washing the ocular surface with PBS as described above and pooling samples from 10 mice per group. Total protein concentration of pooled ocular surface washes was determined with a BCA assay (Pierce, Rockford, IL), and equivalent amounts of protein resolved by SDS-PAGE (Tris-HCl Ready Gel 10%, BioRad, Hercules, CA) under denaturing conditions. Proteins were transferred to nitrocellulose by electroblotting (180 mAmps for 2 h) in transfer buffer (25 mM Tris, 190 mM glycine, and 10% (v/v) methanol). Membranes were blocked with a solution of 10% dry-skim milk suspended in PBS containing 0.1% Tween-20 (PBS-T) for 3 h at room temperature. Primary antibody solution contained rabbit anti-SP-D IgG (generous gift of Dr. Samuel Hawgood, University of California, San Francisco) diluted 1∶750 in PBS-T (4°C for 10 h). After washing thoroughly with PBS-T, a secondary antibody solution was used consisting of anti-rabbit IgG-horseradish peroxidase conjugate (BioRad) diluted 1∶3,000 in PBS-T (room temperature for 1 h). After PBS-T washing, membranes were visualized with a chemiluminescence substrate (Western Lighting ECL, Perkin Elmer, Waltham, MA) and imaging system (FluorChem, Alpha Innotech, Santa Clara, CA).

### Statistical Analysis

Tear volume analysis involved repeated samples on the same animals were thus compared using a parametric repeated measures ANOVA and Bonferroni post-test. Viable bacterial counts were compared using a nonparametric Mann-Whitney t-Test. Incidences of bacterial infections were compared by Chi square analysis. P values<0.05 were considered significant. All experiments were repeated at least once.

## Results

### EDE Mice Show Similar *P. aeruginosa* Tissue Colonization and Pathology to Controls

**Figure 1:**
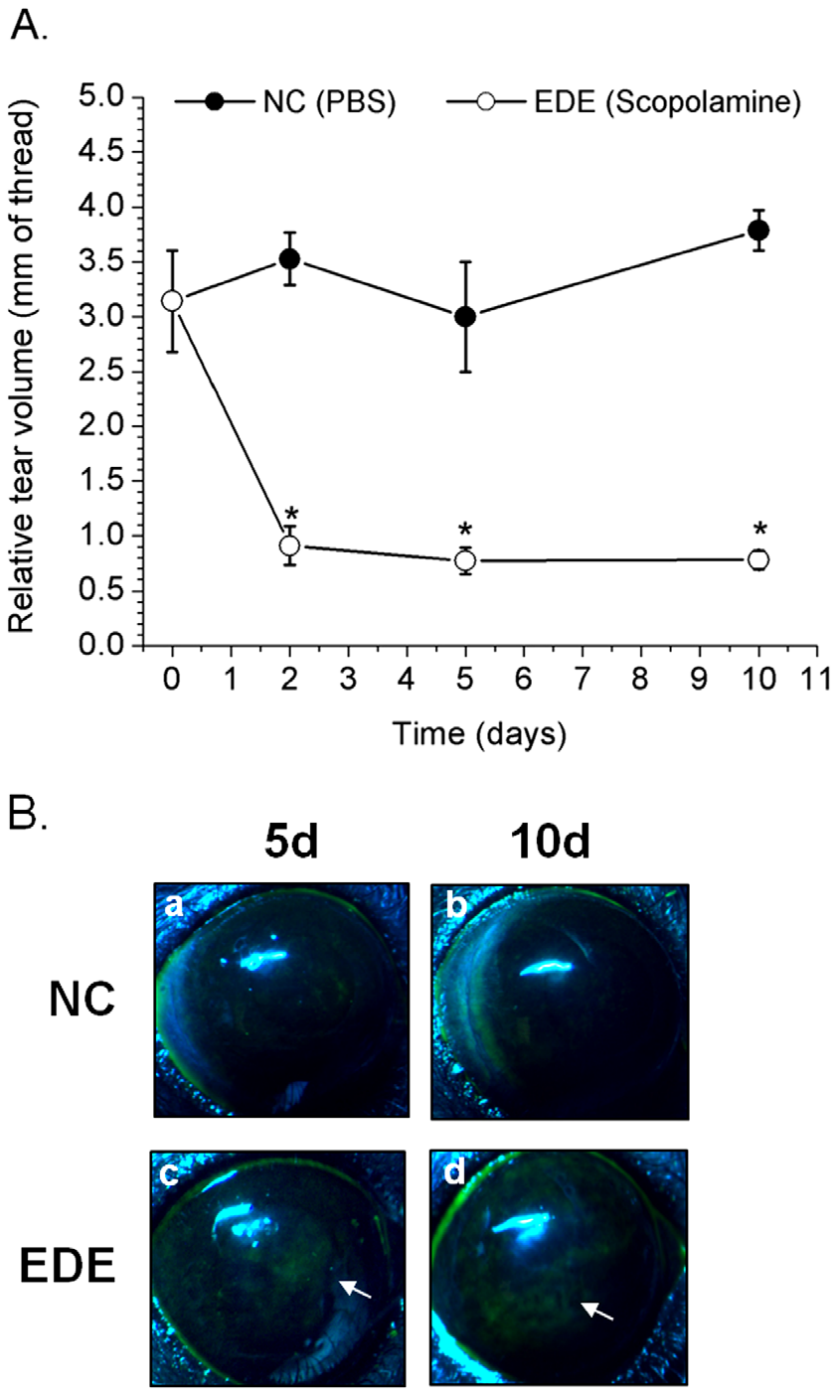
Induction of experimental dry eye. Tear volumes (A) and fluorescein staining (B) in the eyes of C57BL/6 mice under experimental dry eye (EDE) conditions versus normal controls (NC). (A) EDE resulted in significant decreases in tear volume after 2 days. Tears were collected from the lateral canthus using cotton thread and reported as millimeters of wetted thread. Data are expressed as the mean +/− standard deviation per group from three independent experiments (≥3 mice per group for each experiment). * Denotes significance differences between treatment groups (determined with a parametric repeated measures ANOVA and Bonferroni post-test), p<0.001 in each instance). (B) Corneal integrity was assessed by fluorescein staining in EDE mice or normal controls after 5 or 10 days. Eyes were examined under blue light illumination at 20-x magnification. Photographs are representative of three independent experiments (≥3 mice per group for each experiment). Control mouse eyes are shown in the upper panels (a, b), EDE mouse eyes are shown in the lower panels (c, d). Arrows denote regions of fluorescein staining on the ocular surface.

EDE was induced in C57BL/6 mice for 5–10 days as described previously [26], [37]. This model is known to induce damage in the lacrimal gland, cornea, and conjunctiva without grossly affecting other mucosal and non-mucosal tissues [39], [40]. As previously reported [37], we observed a significant decrease in tear volume (∼70%) compared to control mice within the first 2 days of administering scopolamine and evaporative air drafts, and this decrease was maintained for 10 days (Fig. 1A). EDE mice also showed fluorescein penetration of the cornea after 5 days, which intensified up to 10 days with continued scopolamine and air draft exposure (Fig. 1B, lower panels). Control mice did not show significant fluorescein staining (Fig. 1B, upper panels). After 10 days of EDE, mice were topically challenged with *P. aerugin*osa strain PAO1 (∼10^9^ cfu) without other prior injury to the cornea. Dry eye conditions were maintained and ocular pathology observed for up to 4 days (96 h) post-inoculation (pi). After 4 days, there were relatively few instances of ocular pathology. Less than 15% of challenged corneas displayed pathology, and there was no significant difference in disease incidence between EDE and control mice [EDE 14% versus control 10%; p-value (Chi square) = 0.75] (Table 1). When present, corneal pathology manifested as focal or punctate opacities observed within 24 h of challenge and varied from mild to moderate severity with disease scores of 4, 5, and 6, Table 1) after 4 days.

**Table 1:** Incidence and severity of *P. aeruginosa* infections in EDE mice.

Treatment Group	Incidence of Pathology	Pathology Scores	Chi-square Value
Control	1 (10 mice)	6	0.75
EDE	2 (14 mice)	4, 5	

C57BL/6 mice were exposed to EDE or control conditions for 10 d prior to topical challenge with 10^9^ cfu of *P. aeruginosa* strain PAO1. Mice were monitored for corneal infiltrates, opacities, and changes in epithelial surface regularity. Pathology was graded at 96 h post-inoculation (see Materials and Methods). Incidences of pathology in EDE and control groups were not significantly different (Chi-square analysis). Data is representative of two independent experiments.

### Enhanced *P. aeruginosa* Clearance From Ocular Surface Washes of EDE Mice

**Figure 2:**
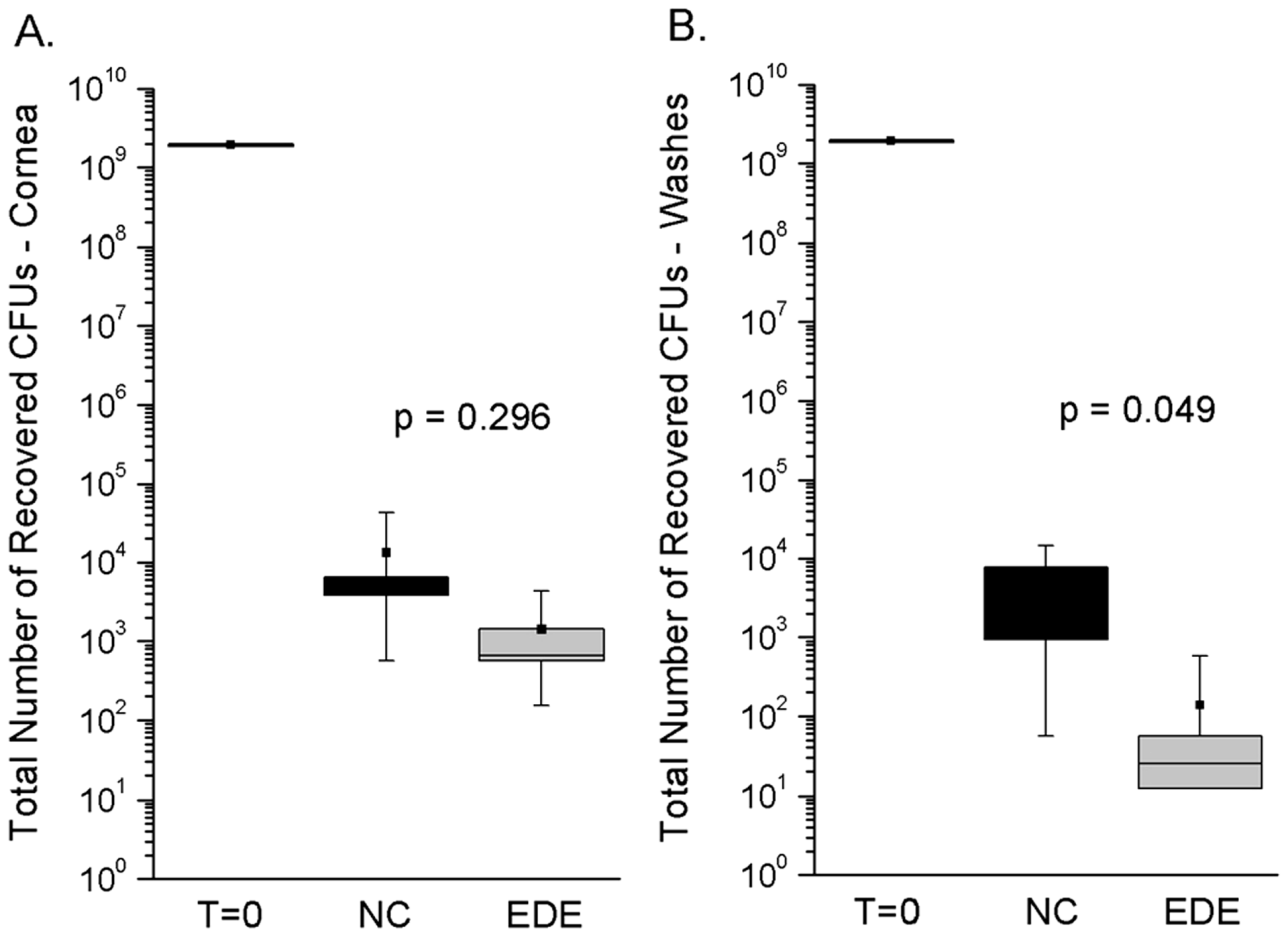
Ocular clearance of *P.* *aeruginosa* in EDE. Levels of viable *P. aeruginosa* (cfu) in corneal homogenates (A) or ocular surface washes (B) of C57BL/6 EDE mice compared to normal controls (NC) at 6 h post-inoculation with 10^9^ cfu of *P. aeruginosa* strain PAO1 (T = 0). EDE was induced for 5 days prior to bacterial inoculation. Bacteria were rapidly cleared from the murine ocular surface of both groups of mice after 6 h. Similar bacterial levels were found in corneal homogenates (A), but fewer bacteria were recovered from the ocular surface washes of EDE mice compared to controls (p = 0.049, Mann-Whitney test) (B). Data are representative of three independent experiments (≥5 animals per group in each experiment). Data for each sample are shown as the median (black square) with upper and lower quartiles (boxed area), and range of the data (error bars).

The initial clearance of *P. aeruginosa* from the ocular surface was assessed by measuring viable bacteria in corneal homogenates and ocular surface washes of EDE mice compared to normal controls at 6 h post-inoculation. EDE was induced for 5 days before bacterial inoculation. The majority of the *P. aeruginosa* inoculum (>99.9%) was rapidly cleared from the ocular surface (corneal homogenates and ocular surface washes) of both EDE and control mice after 6 h (Fig. 2). This was consistent with our previous studies using a similar “null infection” model [36], [41]. EDE and control mice were not significantly different with respect to bacterial numbers in corneal homogenates after 6 h (Fig. 2A). However, there was a significant reduction (∼50-fold) in the number of viable bacteria recovered from ocular surface washes of EDE mice compared to controls after 6 h (Fig. 2B, p = 0.049, Mann-Whitney test) showing that EDE enhanced the ocular clearance of *P. aeruginosa*.

### Increased Expression of SP-D in Ocular Surface Washes of EDE Mice

**Figure 3:**
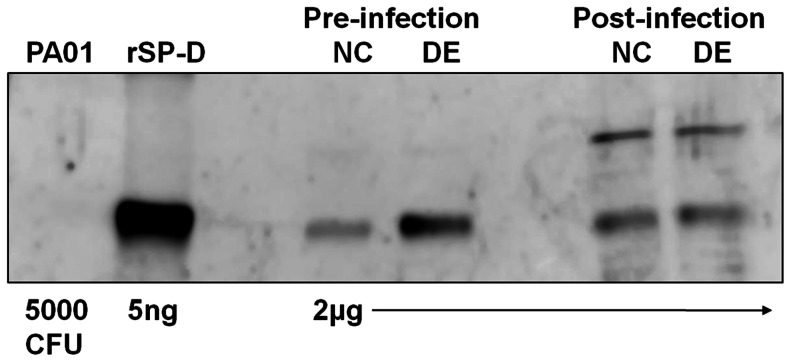
SP-D expression in EDE before and after *P.* *aeruginosa* challenge. Western immunoblot blot analysis of SP-D expression in pooled ocular surface washes from EDE and control mice (10 mice per group) after 5 days EDE induction, and before and 6 h after inoculation with *P. aeruginosa* strain PAO1 (10^9^ cfu). To normalize for differences in tear volume, equivalent amounts of protein (2 µg) were used in the analysis (BCA protein assay). Purified recombinant SP-D (rSP-D, ∼43 kDa monomer), and a relevant number of bacteria suspended in PBS (5×10^3^ cfu, see Fig. 2B), were included as positive and negative controls, respectively. SP-D expression in ocular surface washes was increased under EDE conditions before bacterial inoculation. The experiment was repeated once.

We have previously shown that SP-D, a member of the collectin family of innate defense molecules, is present in tear fluid and the corneal epithelium and plays a role in ocular defense against *P. aeruginosa* [34], [36]. Thus, SP-D levels were assessed in EDE mice and controls after 5 days of EDE induction, and before and after (6 h) inoculation with 10^9^ cfu PAO1. To account for differences in tear volume, equivalent amounts of total protein from each sample were used for analyses. EDE mice showed increased expression of SP-D in ocular surface washes compared to normal controls prior to bacterial inoculation (Fig. 3). This difference was not seen the post-inoculation ocular surface washes, although the latter samples did contain an additional form of SP-D which we have also observed in a previous study [34]. The antibody against SP-D did not react with bacteria alone. These data show that EDE also increases expression of SP-D at the murine ocular surface.

### EDE Increases *P. aeruginosa* Corneal Colonization in SP-D Deficient Mice

**Figure 4:**
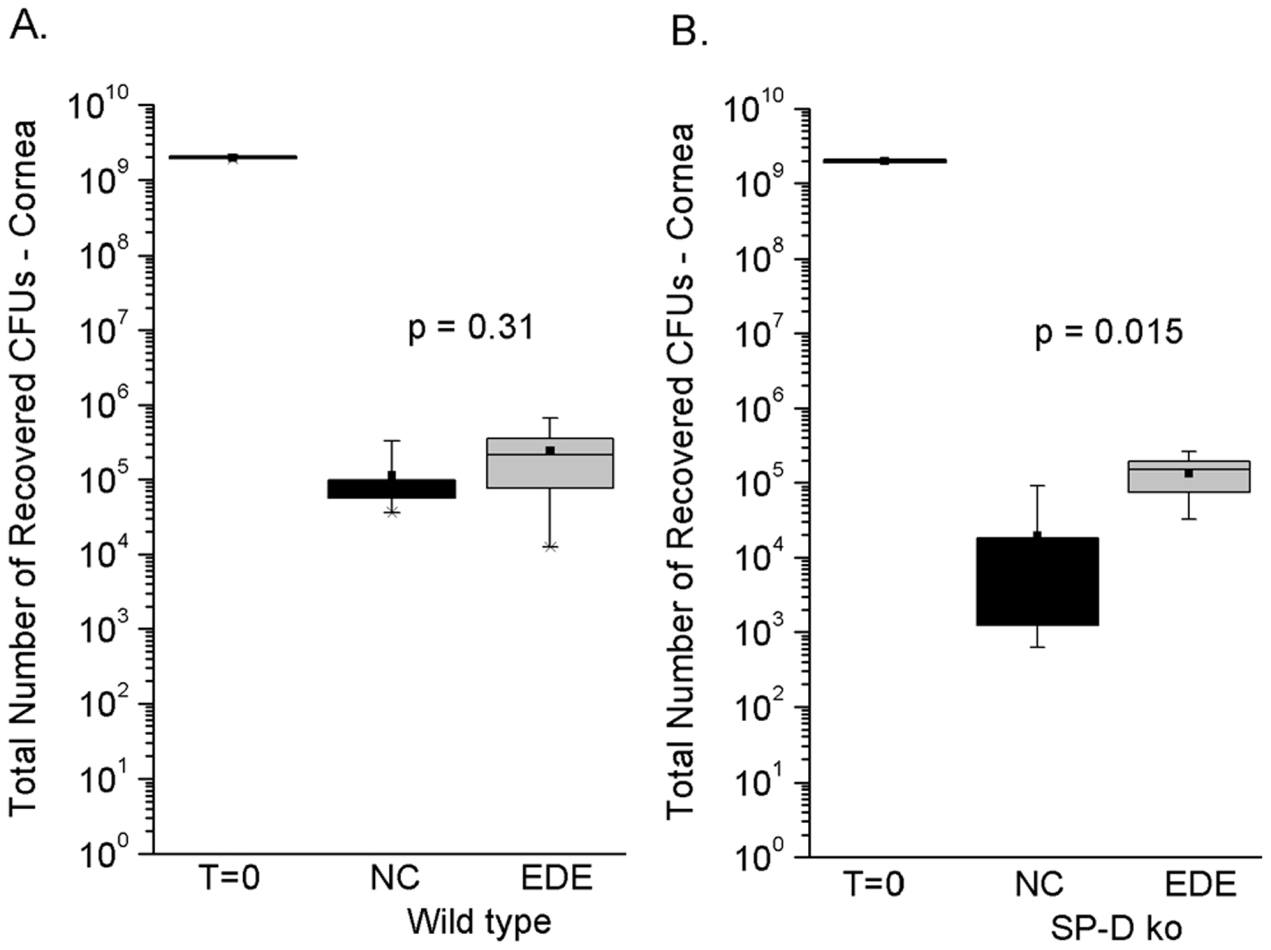
Effect of EDE on *P.* *aeruginosa* corneal colonization in SP-D knockout mice. Corneal colonization by *P. aeruginosa* in normal Black Swiss mice (A) or SP-D deficient age/sex-matched Black Swiss mice (B) under normal (NC) and experimental dry eye (EDE) conditions. After 5 days EDE induction, otherwise uninjured corneas were challenged with 10^9^ cfu of *P. aeruginosa* strain PAO1 (T = 0). EDE did not affect bacterial colonization in wild-type mice after 6 h. However, EDE in SP-D knockout mice (*sp-d* −/−) resulted in a ∼5-fold increase in corneal colonization after 6 h. Data shown is representative of two independent experiments with SP-D-deficient Black Swiss mice (n≥5 animals per group). P values were obtained using the Mann-Whitney Test. Data for each sample are shown as the median (black square) with upper and lower quartiles (boxed area), and range of the data (error bars).

Although EDE enhanced SP-D expression in ocular washes of normal mice prior to bacterial challenge, normal mice had previously shown no difference in ocular colonization between normal and EDE conditions. Thus, SP-D deficient (*sp-d* −/−) mice were tested for *P. aeruginosa* corneal colonization under EDE conditions. Since *sp-d* gene knockout mice were available in a Black Swiss background, a control colonization experiment was done using wild-type Black Swiss mice. EDE did not affect corneal colonization by *P. aeruginosa* in wild-type Black Swiss mice (Fig. 4A), consistent with our earlier results from wild-type C57BL/6 mice (Fig. 2A). SP-D deficient mice were also exposed to EDE or normal conditions (NC) for 5 days then challenged with 10^9^ cfu of *P. aeruginosa* strain PAO1. A significant increase in bacterial corneal colonization (∼5-fold) was observed in SP-D deficient mice under EDE compared to normal conditions at 6 h (Fig. 4B). Thus, without SP-D, EDE is associated with increased *P. aeruginosa* corneal colonization.

## Discussion

Dry Eye Disease is denoted by low tear volumes and inflammatory damage to the conjunctiva and/or cornea [42]. As such, dry eye disease has the potential to increase susceptibility to infection. The results of the present study, however, show that induction of dry eye disease in a murine experimental model (EDE) did not increase corneal susceptibility to *P. aeruginosa* infection with minimal pathology observed in both normal and dry eye mice. The data also showed that EDE resulted in an increase in surfactant protein-D expression at the ocular surface (ocular surface washes) before bacterial inoculation, and this correlated with increased bacterial clearance from the tears (ocular surface washes) of EDE mice. While corneal colonization was unaffected by dry eye disease in wild-type mice, our data showed that *sp-d* gene knockout mice showed increased corneal colonization under EDE conditions. Together these data show that dry eye disease does not compromise ocular defenses against *P. aeruginosa* infection, and suggest that SP-D contributes to ocular defense against infection under EDE conditions.

Upregulation of SP-D in ocular surface washes in response to dry eye conditions may reflect a compensatory innate defense response. This would be consistent with previous studies which have suggested that other ocular innate defenses are upregulated in patients with dry eye disease including membrane-associated mucins (e.g. MUC1) [21], [43] and human beta-defensins [18], [19]. SP-D has antimicrobial, aggregative and opsonizing properties against *P. aeruginosa*, it is present in tear fluid, inhibits *P. aeruginosa* internalization by corneal epithelial cells, and it promotes ocular clearance of *P. aeruginosa* in vivo [34], [36], [44]–[47]. Each of these protective effects could help provide enhanced defense of the ocular surface from infection during dry eye disease, more so if combined with antimicrobial and anti-adhesive actions of other innate defenses, e.g. defensins and mucins respectively [41], [48], [49]. In our study, enhanced SP-D expression in ocular surface washes of dry eye mice correlated with reduced numbers of viable bacteria in those washes. However, further studies will be needed to determine the relative role(s) of SP-D, and other ocular surface antimicrobial defenses that are likely to be upregulated, in removing *P. aeruginosa* from the ocular surface under the dry eye conditions in this model.

The mechanism for SP-D upregulation in ocular washes of EDE mice is not yet known. We have previously shown that *P. aeruginosa* flagellin and LPS antigens can each upregulate SP-D production and secretion in corneal epithelial cells, the latter through a mechanism involving JNK [35]. However, upregulation in dry eye mice occurred before bacterial inoculation. Thus, other facets of dry eye disease must trigger increased SP-D expression at the ocular surface. Mechanisms of SP-D expression and upregulation in various mammalian cells are complex and incompletely understood [35], [50], [51]. However, dry eye disease in murine models or humans is known to involve increased expression of proinflammatory mediators such as IL-1β, IL-6, IL-8, and involve MAP kinase signaling proteins including JNK [22], [52]–[54]. SP-D is also known as an immuno-modulator with *sp-d* knockout mice showing enhanced inflammatory-mediated tissue pathology in the cornea and other animal infection models [47], [55]. It is possible, therefore, that increased SP-D expression in EDE occurs in response to ocular inflammation, and that it functions to modulate those responses and protect against bacterial challenge.

Two different mouse strains were used in this study (C57BL/6 and Black Swiss). We are unaware of any differences in SP-D expression between these and other mouse strains. Black Swiss mice show a bias towards Th2 responses, and a SP-D knockout mouse in that strain could show greater Th2 responsiveness, as shown in models of lung allergy [56]. It has been also shown that BALB/c mice (which also show a T_H_2 bias) produce lower levels of pro-inflammatory cytokines and display less severe changes in goblet cell density under EDE conditions compared to C57BL/6 mice which have a T_H_1 bias [57]. However, further studies will be needed to determine the relationship, if any, between different mouse strains, SP-D expression, and *P. aeruginosa* colonization under EDE conditions.

In conclusion, experimental dry eye mice were not inherently more susceptible to *P. aeruginosa* infections than controls. These animals were able to displace bacteria from the ocular surface and displayed relatively low incidence rates of mild to moderate pathology, comparable to normal controls. Although dry eye disease in this model can promote desquamation of the superficial corneal epithelial cells, decrease the relative number of intercellular tight junctions [25], [26], it is recognized that other protective defenses can be upregulated in dry eye including pro-inflammatory mediators, defensins and mucins. Our data shows that SP-D is also upregulated in dry eye conditions, and may contribute to ocular resistance to infection during desiccating stress.
